# Hypoxia-Induced Epithelial-Mesenchymal Transition in Cancers: HIF-1α and Beyond

**DOI:** 10.3389/fonc.2020.00486

**Published:** 2020-04-08

**Authors:** Shing Yau Tam, Vincent W. C. Wu, Helen K. W. Law

**Affiliations:** Department of Health Technology and Informatics, Faculty of Health and Social Sciences, The Hong Kong Polytechnic University, Kowloon, Hong Kong

**Keywords:** cancer, epithelial-mesenchymal transition, HIF-1α, hypoxia, metastasis, signaling pathway

## Abstract

Metastasis is the main cause of cancer-related mortality. Although the actual process of metastasis remains largely elusive, epithelial-mesenchymal transition (EMT) has been considered as a major event in metastasis. Besides, hypoxia is common in solid cancers and has been considered as an important factor for adverse treatment outcomes including metastasis. Since EMT and hypoxia potentially share several signaling pathways, many recent studies focused on investigate the issue of hypoxia-induced EMT. Among all potential mediators of hypoxia-induced EMT, hypoxia-inducible factor-1α (HIF-1α) has been studied extensively. Moreover, there are other potential mediators that may also contribute to the process. This review aims to summarize the recent reports on hypoxia-induced EMT by HIF-1α or other potential mediators and provide insights for further investigations on this issue. Ultimately, better understanding of hypoxia-induced EMT may allow us to develop anti-metastatic strategies and improve treatment outcomes.

## Introduction

Metastasis is the major cause of cancer-associated deaths (1). It is a sequential event of uncontrolled cell proliferation, angiogenesis, detachment, motility, invasion into bloodstream, settle in the microvasculature, and finally extravasation from the blood vessel and proliferation in secondary sites. It is a complicated process involving multiple genes and signaling pathways for each step (2–4). Although much of the exact mechanism remains unknown, epithelial-mesenchymal transition (EMT), which is a cellular process that enables a polarized epithelial cell to undergo changes to be a mesenchymal cell phenotype, has been regarded as an important event for metastasis (4). Apart from EMT, hypoxia, which is cell having a lower oxygen tension than normal condition, is a common phenomenon in most solid tumors (5). Hypoxia could trigger various signaling pathways which may lead to adverse clinical outcomes in cancer including higher invasiveness and tendency to metastasize (5–7). Studies in the field of cancer biology have linked these two important tumorigenesis events together when unraveling the process of metastasis (8). Among different hypoxia-related pathways, hypoxia-inducible factor-1α (HIF-1α) has been studied extensively (9). In this review, we summarize previous researches and recent findings of the effect of hypoxia on EMT induction with emphasis in various hypoxia-related mediators.

## EMT in Cancer

EMT is involved during the implantation of the embryo and the initiation of placenta formation (Type 1), inflammation and fibrosis (Type 2), and in the change of primary epithelial cancer cell to invasive and metastatic mesenchymal cell (Type 3) (10). Type 3 EMT (hereby referred as EMT) comprises activation of transcription factors, expression of specific cell surface proteins, reorganization and expression of cytoskeletal proteins, production of extracellular matrix (ECM)-degrading enzymes and changes in the specific microRNA (miRNA) expressions (10). This leads to increased invasiveness, migratory capacity, production of ECM components and resistance to apoptosis of cancer cells (10). Furthermore, EMT could affects the immune cell functions in the tumor microenvironment and promotes an immunosuppressive tumor microenvironment to escape immune surveillance by immune cells (11).

Epithelial cells are held together by various cell adhesion molecules including claudins and E-cadherin for attachment to both the basement membrane and adjacent cells and maintenance of epithelial phenotype. The loss of function or expression of E-cadherin and tight junction proteins, and also the increase of mesenchymal markers including vimentin, fibronectin, and N-cadherin, have been considered as the main molecular events of EMT (12). Cadherins are transmembrane components of the adherens junction, which play important role in cell-cell adhesion and actin cytoskeleton (13). E-cadherin is pre-dominantly expressed by normal epithelial tissues. However, many epithelial cancer cells have reduced E-cadherin expression and the loss of E-cadherin is correlated to poor prognosis in a variety of cancers (14). While N-cadherin is typically expressed in mesenchymal cells, which are more spindle-shaped and less polarized than epithelial cells (13). Hence, the transition from E-cadherin to N-cadherin is considered as the major process in EMT induction. Nonetheless, the disintegration of adherens junctions between cells changes the cytoskeletal composition and cell polarity to a more spindle-shaped form. In cancer cells undergoing EMT, the actin cytoskeleton is reorganized from cortical thin bundles into thick contractile stress fibers at the ventral cell surface (15). The monomers of actin, i.e., globular-actin (G-actin), polymerize to form filamentous-actin (F-actin) to start the formation of various migratory protrusions including podosomes, invadopodia, filopodia, and lamellipodia. This process is known as dynamic actin reorganization (16). It is a prerequisite for the morphology change, migration and invasion of cancer cells (16, 17). Another protein, vimentin, is a Type III intermediate filament protein that is a major cytoskeletal component of mesenchymal cells (18). Whereas, fibronectin is a stromal ECM protein that binds to integrin receptors to link the ECM with cytoskeleton (19). The up-regulations of these mesenchymal markers also mark the EMT induction process. Moreover, cells undergoing EMT could degrade and invade basal extracellular matrix by matrix metalloproteinases (MMPs) (11).

There are several EMT transcription factors including the zinc-finger binding transcription factors: Snail1 (Snail) and Snail2 (Slug), zinc finger E-box-binding homebox 1/2 (ZEB1/2), TWIST, and lymphoid enhancer-binding factor-1 (LEF-1). They bind to the promoter region of cell adhesion genes and repress their transcription. The reduced cell adhesion initiates EMT. These core EMT transcription factors have non-redundant functions yet they may cooperate to promote EMT (20). Snail and Slug bind to the promoter of cadherin-1 (CDH1), which encodes E-cadherin, to repress its transcription. The other molecules, ZEB1 and ZEB2, mediate the bipartite E-box regions of DNA for flanking the CDH1 gene, resulting in E-cadherin repression. Both Twist-related protein 1 (TWIST1) and Twist-related protein 2 (TWIST2) belong to the basic helix-loop-helix (bHLH) transcription family. They also flank the CDH1 gene to repress E-cadherin. In addition, it has been reported that TWIST1 binds with Slug promoter to stimulate EMT in human mammary cells (21). LEF-1 could also directly repress E-cadherin and induce EMT (22). Its overexpression in colon carcinoma cell lines could promote EMT by nuclear β-catenin activation (23). In general, these factors are usually associated with poor prognosis in different types of cancer (24–35).

Multiple signaling pathways that are involved in these EMT-inducing factors have been reviewed recently (12). Furthermore, miRNA-transcription factor regulatory circuits, along with long non-coding RNAs, have also been proposed recently for complex control of EMT process (11, 36). In this review, we focus on the pathways related to hypoxia.

## Tumor Hypoxia

Hypoxia is a common phenomenon in most solid tumors (5). Even though tumors are developed by clonal expansion, the cells are in different stages of maturation and differentiation. Tumor cells are also arranged in different geometry. Therefore, each individual tumor is a heterogeneous population of cells and each individual tumor cell has its own microenvironment (37). Although tumor cells can promote angiogenesis that stimulate the growth of endothelial cells from neighbor blood vessels for the supply of nutrients, over-population, increase in oxygen diffusion distances of cells, anarchic tumor vasculature with irregular blood flow and low oxygen diffusion are common causes of poor oxygenation (37–39). In addition, hypoxia could be more prominent due to tumor-induced or treatment-induced anemia and low hemoglobin levels in blood (39). In normal tissues, the oxygen tension (pO_2_) is normally 10–80 mmHg, while tumors often contain low oxygen concentration regions of severe hypoxia (<0.5 mmHg) and intermediate hypoxia (0.5–20 mmHg) (5). Hypoxia could pose a variety of adverse clinical outcome during the treatment of cancer. It has been reported to increase radioresistance at pO_2_ level of <1–10 mmHg, genomic instability, angiogenesis, vasculogenesis, invasiveness, boosted stem cell properties. Most importantly, cells under hypoxia may have higher tendency to metastasize and improved survival in nutrient deprived environment (5, 7).

Hypoxia-inducible factors (HIFs) are the major transcriptional regulators in response to hypoxia, which consist of an oxygen-regulated HIF-α subunit (HIF-1α or HIF-2α) dimerizing with HIF-1β in hypoxia. It activates target gene transcription with CREB-cAMP-response element binding protein (CBP) in hypoxia responsive elements (HRE). In normoxia, HIF-1α is hydroxylated at proline 402 and 531 while HIF-2α is hydroxylated at proline 405 and 531 by HIF-α prolyl hydroxylases (PHDs) and factor inhibiting HIF (FIH) proteins within its oxygen-dependent degradation domain (ODD) of PHDs. This process regulates the binding of von Hippel-Lindau (VHL) tumor suppressor E3 ligase for Lys48-linked polyubiquitination of HIF-α and finally results in proteasomal degradation. Whereas, FIH hydroxylates HIF-1α and HIF-2α at asparagine 803 and 847 within the C-terminal transactivation domain, respectively. This action blocks HIF interaction with p300 or CBP and prevents transcription of target genes (40–42).

HIF-1α and HIF-2α have distinct physiological roles though they are similar in overall amino acid sequence, domain structure and activation mechanisms (43). HIF-1α is usually up-regulated more prominently in shorter time interval (2–24 h) and lower oxygen level (<0.1% O_2_) whereas HIF-2α is usually up-regulated in a higher oxygen level (<5% O_2_) with longer maintenance time (48–72 h) in some cell lines (39, 40). HIF-1α regulates a variety of tumor processes for adaptation, such as metabolism, erythropoiesis, angiogenesis, invasion, cell survival and proliferation (40, 44). HIF-1α could regulate various EMT transcription factors, histone modifiers [e.g., histone lysine-specific demethylase 4B (KDM4B)], enzymes [e.g., lysyl oxidase (LOX), MMP1, MMP3], chemokine receptors 1 and 4 (CX3CR1, CXCR4), adhesion molecules [e.g., angiopoietin-like 4 (ANGPTL4), L1 cell adhesion molecule (L1CAM)], and miRNA targets to promote metastasis (45). Its expression was associated with poor treatment outcome in different types of cancer (44, 46, 47).

Apart from hypoxia-induced HIF-1α activation, HIF-1α could be controlled by oxygen-independent oncogenic regulation, which includes growth factor signaling pathways such as phosphatidylinositol-3-kinase (PI3K) activation, mouse double minute 2 homolog (Mdm2) pathway and heat shock protein 90 (Hsp90) (48). In addition, HIF-1α activation is associated with the Warburg metabolites including glucose transporters and glycolytic enzymes (49). Furthermore, reactive oxygen species (ROS) could stabilize HIF-1α under normoxia via several proposed models (50).

Though HIF-1α has been intensively researched, HIF-2α was less studied. A recent study by Li et al. (51) found that HIF-2α was significantly expressed in the cancer stem cell population but not in other tumor cells. Moreover, HIF-2α was proposed to promote stem cell marker expression, a stem cell phenotype and tumorosphere formation in hypoxic conditions (38). Therefore, HIFs are required for cancer stem cell survival and tumor progression (38). In fact, HIF-1α and HIF-2α may have antagonistic roles in some cellular functions including cell growth. For example, HIF-1α promoted the growth of SW-480 colon cancer cells while HIF-2α suppressed the tumor growth (41). It is important not to generalize because HIF-1α and HIF-2α may have different effect in other tumor cell lines (43). Recent studies also showed HIF-1α and HIF-2α regulate various non-coding RNAs for facilitating tumorigenesis (52).

## HIF-Mediated EMT

**Figure 1:**
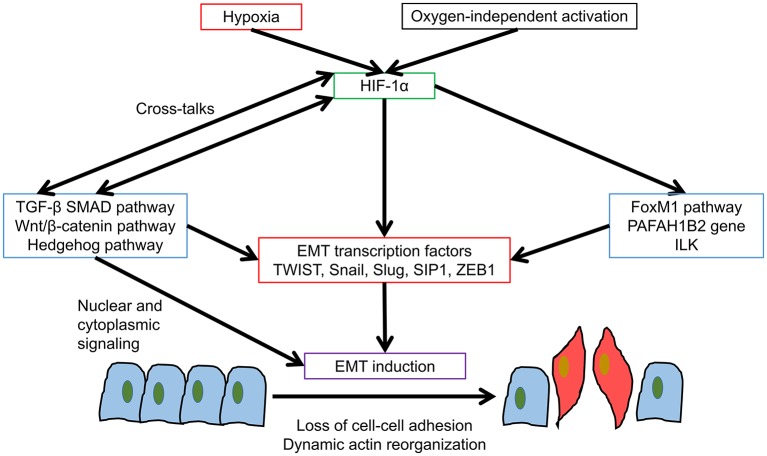
HIF-1α mediated EMT. HIF-1α promotes EMT induction in various cancer types by multiple ways. Various pathways promote EMT induction, resulting in loss of cell-cell adhesion and dynamic actin reorganization.

Initially, both EMT and hypoxia are considered as separate events promoting invasion and metastasis of various types of cancer. Recently, the term hypoxia-induced EMT has been proposed because the signaling pathways are inter-related. Among all the signaling pathways involved in hypoxia, the HIF pathway was proposed to be the most important one for hypoxia-induced EMT though it may also be regulated by oxygen-independent mechanisms (Figure 1). In earlier studies, HIF-1α has been linked with transforming growth factor β (TGF-β) activation in hepatocyte during liver fibrosis and human umbilical vein endothelial cells (53, 54). TGF-β can also suppress both mRNA and protein expressions of PHD2 and consequently increases HIF-1α stability (55). TGF-β is the most studied EMT signaling pathway, which includes the HIF-1α related mothers against decapentaplegic homologs (SMAD) signaling and non-SMAD signaling. For SMAD signaling, the phosphorylation of TGF-βRI activates SMAD signaling after binding to TGF-βRII and TGF-βRIII. Then the oligomerization of SMAD2/3 with SMAD4 and the nuclear import of the R-SMAD/SMAD4 complex are enabled for binding regulatory elements inside the nucleus and inducing the transcription of EMT associated genes. While non-SMAD signaling involved various mitogen-activated protein kinases (MAPKs) and PI3K- protein kinase B (Akt)- mechanistic target of rapamycin (mTOR), which will be introduced in later parts of this review (56). HIF-1α was found to regulate TGF-β-SMAD3 pathway in breast cancer patients (57). Apart from the crosstalk between HIF-1α and TGF-β, another important EMT pathway Wnt/β-catenin could also enhance hypoxia-induced EMT by potentiating with HIF-1α signaling in hepatocellular carcinoma (58). Moreover, HIF-1α is linked to the expression of immunosuppressive molecules in tumor cells, which could potentiate EMT induction through TGF-β signaling (59, 60). EMT could in turn elicit multiple immune-regulatory effects causing natural killer (NK) and T-cell apoptosis and increase of regulatory T and B cells (61). Furthermore, HIF-1α was found to mediate hedgehog signaling for EMT and invasion in pancreatic cancer cells and the silencing of HIF-1α would reverse hypoxia-induced hedgehog signaling activation (62). Therefore, there were definitive cross-talks between HIF-1α and other EMT signaling pathways yet the relationship could be tumor-type and context-dependent.

Aside from the studies concerning the cross-talks between HIF-1α and other EMT pathways, more researches have focused on HIF-1α modulation of various EMT transcription factors including TWIST, Snail, Slug, SIP1, and ZEB1 (Table 1). For HIF-1α-TWIST interaction, HIF-1α could bind directly to TWIST by HRE in the TWIST proximal promoter in hypopharyngeal and breast cancer cell lines. It also promoted metastasis and the over-expression of TWIST was essential for HIF-1α-mediated EMT and non-redundant when compared with other EMT inducers such as Snail (63). The co-expression of HIF-1α, TWIST, and Snail in primary tumors of head and neck cancer patients correlated with the poorest prognosis (63). The up-regulation of TWIST by HIF-1α was also found among clinical samples of ovarian epithelial cancers and was associated with lower overall survival rate (26).

**Table 1:** HIF-1α-EMT transcription factors association studies in different cancer types.

**EMT transcription factor**	**Cancer type (cell line, samples studied)**	**References**
TWIST	Hypopharyngeal cancer (FaDu) Breast cancer (MCF-7)	(63)
	Ovarian epithelial cancer (Clinical samples)	(26)
Snail	Hepatocellular carcinoma (HepG2 and SMMC-7721)	(34)
	Lung adenocarcinoma (A549)	(64)
	Renal clear-cell carcinoma (786-O)	(65)
Slug	Head and neck squamous cell carcinoma (UM-SCC1, UM-SCC23, Clinical samples)	(33)
	Lung adenocarcinoma (A549)	(64)
	Prostate cancer (LNCaP)	(66)
	Pancreatic ductal adenocarcinoma (AsPC-1, BxPc-3, Capan-1, Capan-2 and MIA-PaCa2)	(67)
SIP1	Renal clear-cell carcinoma (786-O)	(65)
ZEB1	Pancreatic ductal adenocarcinoma (AsPC-1, BxPc-3, Capan-1, Capan-2 and MIA-PaCa2)	(67)
	Colorectal cancer (HT-29 and HCT-116)	(68)
	Bladder cancer (T24-P, T24-L, Clinical samples)	(69)
	Glioblastoma (SNB78 and U87)	(70)
	Pancreatic cancer (PANC-1 and SW-1990)	(71)

For HIF-1α-Snail interaction, HIF-1α first activates histone deacetylase 3 (HDAC3). HDAC3 could then bind to the promoters of CDH1 and junction plakoglobin (JUP) and eventually promote transcription of Snail (12, 72). In an *in silio* analysis by Luo et al. (73), HIF was found to bind with a putative HRE within minimal Snail promoter of mouse, demonstrating possible direct interaction between HIF and Snail. HIF-1α-induced Snail activation was found in liver and lung cancers (34, 64). Zhang et al. (34) found both HIF-1α and Snail overexpression were correlated with pathological classification, TNM staging, and tumor volume in hepatocellular carcinoma patients. The disease-free survival was also significantly shorter in HIF-1α positive group than HIF-1α negative group. This study also showed the elevation of Snail mRNA expression level after HIF-1α stabilization accompanied with E-cadherin repression plus vimentin and N-cadherin up-regulation. Meanwhile, in a shorter path, HDAC3 also regulates the formation of histone methyltransferase complexes by WD repeat-containing protein 5 (WDR5) recruitment to induce vimentin and N-cadherin expression (12, 72). As a chromatin modifier, HDAC3 could directly deacetylate histone H3 Lys4 acetylation (H3K4Ac) for promotion of EMT marker genes and indirectly increased the levels of histone H3 Lys4 di/trimethylation (H3K4me2/3) through WDR5, yet the exact molecular mechanisms remained to be explored (74).

For another important zinc-finger binding transcription factor Slug, HIF-1α was associated with its expression in head and neck squamous carcinoma, lung, and pancreatic cancer cells (33, 64, 66, 67). Similar to Snail, Slug was also suggested to contain HRE in its promoter for direct interaction between HIF-1α and Slug (75). SIP1 activation, together with Snail activation and E-cadherin repression, were found to be HIF-1α-mediated in VHL^−/−^ renal clear-cell carcinoma cell line (65). The reintroduction of wild-type VHL could suppress SIP1 and Snail but not Slug, and removed the suppression of E-cadherin (65).

HIF-1α could bind directly to the proximal promoter of ZEB1 via HRE in colorectal cancer cells (68). Additionally, this research group demonstrated the importance of ZEB1 in HIF-1α induced metastasis with higher percentage of HIF-1α and ZEB1 positive staining and lower percentage of E-cadherin positive staining in patients' metastatic lymph nodes compared with primary colorectal cancer tissues (68). The influence of HIF-1α on ZEB1 was also evaluated among bladder cancer (69), glioblastoma (70) and pancreatic cancer cells (67, 71). Joseph et al. (70) has evaluated HIF-1α but not HIF-2α up-regulated ZEB1 under hypoxia in glioblastoma cells.

HIF-1α may also act on some EMT transcription factors indirectly through FoxM1 signaling pathway in prostate cancer cell lines (76) and through PAFAH1B2 gene in pancreatic cancer (77). HIF-1α also facilitated the regulatory loop with integrin-linked kinase (ILK) to promote epithelial-mesenchymal transition in breast and prostate cancer cell lines (78). In the view of previous research findings, it is clear that HIF-1α takes important roles in hypoxia-induced EMT through promoting wide range of EMT transcription factors through multiple signaling pathways in various cancer types. Whereas, for HIF-2α-mediated EMT, researches in this area remained scarce. Notably, HIF-2α could also activate EMT transcription factors including TWIST2 in lung and pancreatic cancer cells and WDR5 for promoting mesenchymal gene expression (72, 79, 80). As HIF-2α has longer activation period at higher oxygen level than HIF-1α, it could be an important mediator of EMT induction at milder hypoxia and thus further studies of HIF-2α-mediated EMT are warranted.

## Hypoxia-Induced Non-HIF EMT Pathways

**Figure 2:**
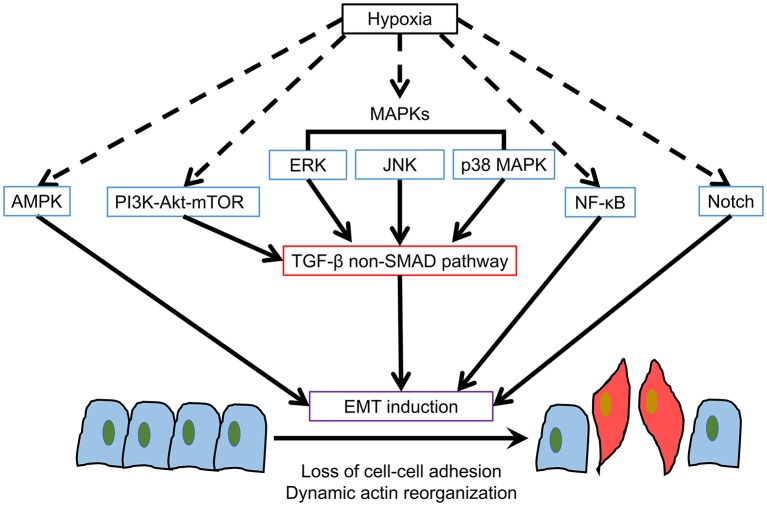
Hypoxia-induced Non-HIF EMT pathways. Apart from HIF-1α, there are several potential hypoxia-induced pathways for EMT induction.

In addition to HIF pathways, other signaling pathways involved in hypoxia may have distinctive characters in inducing EMT [Summarized in Figure 2; (12, 81–85)].

### AMPK

Hypoxia can cause up-regulation of AMP-activated protein kinase (AMPK) as adenosine monophosphate (AMP)/adenosine triphosphate (ATP), or adenosine diphosphate (ADP)/ATP ratios are increased in physiological stresses. AMPK regulates cancer progression, lipid synthesis and oxidation, DNA repair and autophagy (86). Traditionally, AMPK was considered as a metabolic tumor suppressor for tumor cell survival under nutrient depletion (87). However, in the context of AMPK-mediated EMT, contradictive results have been reported. Saxena et al. (88) claimed that AMPK activation by AMPK activator A769662 could increase the expression and nuclear localization of TWIST1 and thus promote EMT induction in breast cancer, melanoma and lung adenocarcinoma cell lines. On the contrary, Chou et al. (89) showed that AMPK activation by another AMPK activator OSU-53 could suppress EMT by modulating the Akt-MDM2-Foxo3 signaling axis. The silencing of AMPK could abrogate the reverse of the mesenchymal phenotype among breast and prostate cancer cell lines. Other researches demonstrated that AMPK could suppress EMT of pancreatic cancer and hepatocellular carcinoma cells (90–92). In the previous researches, specific AMPK activators or inhibitors were used to evaluate AMPK-mediated EMT. The results are controversial, hence, further researches on the character of AMPK in hypoxia-induced EMT are needed.

### PI3K-Akt-mTOR

PI3K-Akt-mTOR is commonly activated in cancer cells. It has important roles in cell proliferation, nutrient uptake, anabolic reactions, and autophagy (93, 94). This pathway can also be activated by hypoxia and interacts with HIF-1α in different cell types (9, 95–97). PI3K-Akt-mTOR has been considered as a mediator of TGF-β signaling through non-SMAD pathway. TGF-β may activate PI3K directly or by activation of epidermal growth factor (EGF) and platelet-derived growth factor (PDGF) receptors in various cell types (12). The mutations in PIK3CA or loss of PTEN is associated with colorectal cancer progression through activation of PI3K-Akt pathway with Akt signaling found to up-regulate Snail and Slug (98). Similar Akt-Snail activation for EMT induction was also found among tongue squamous cell carcinoma cell lines (99). Inhibition of Akt could reverse EMT by restoring E-cadherin in oral squamous cell carcinoma cells (100). In addition, Akt activates both mTORC1 and mTORC2, which are found to induce EMT, motility and metastasis of colorectal cancer by RhoA and Rac1 signaling (101). PI3K-Akt-mTOR could also influence HIF-1α activation as mTOR is an upstream mediator of HIF-1α (97). mTOR regulates translation via phosphorylation of 4E-BP1, which in turn inhibits interaction of eIF4E with translation initiation complex and results in mRNA translation activation and facilitate HIF-1α protein synthesis (102). Although there are plenty of researches demonstrating PI3K-Akt-mTOR mediated EMT, there are no research published to date concerning the hypoxia-driven PI3K-Akt-mTOR in EMT induction.

### MAPKs

MAPKs are evolutionarily conserved kinases for controlling fundamental cellular processes such as cell differentiation, growth, proliferation, apoptosis, autophagy and migration (94, 103). Mainly, three MAPKs extracellular signal-regulated kinase (ERK), c-Jun N-terminal kinase (JNK) and p38 MAPK are hypoxia-related and involved in EMT induction through non-SMAD TGF-β signaling (9, 12, 84, 104). MAPK signaling also plays a role in HIF α-subunit nuclear accumulation and transcriptional activity (102). For ERK pathway, the ERK-associated oncogene RAS overexpression promotes EMT through CyclinD1 and E-cadherin regulation (105). The disruption of ERK1 and ERK2 activation could prevent the delocalization of E-cadherin (104). ERK2 could also regulate EMT by DOCK10-dependent Rac1/FoxO1 signaling (106). For the studies on ERK influence in EMT transcription factors, Slug is a target of RAS pathway among colorectal cancer cell lines with mutant RAS (107). While ZEB1, but not Snail nor Slug, was reported to be the target of ERK for EMT induction in lung cancer cell lines (108). Demonstration of ERK-mediated EMT was also found in pancreatic and prostate cancer cell lines (109, 110). Moreover, FGFR3 and WISP1 overexpression in melanoma could also promote ERK-mediated EMT (111, 112). ERK1 and ERK2, together with other MAPKs JNK and p38 MAPK, were found to stabilize the phosphorylation site of TWIST1 for EMT induction in breast cancer cells (113).

JNK and p38 MAPK mediations of EMT start with the E3 ligase member TRAF6, which in turn activates TGF-βRI for EMT induction (12). In earlier studies, JNK phosphorylation is found to mediate TGF-β1-induced EMT by promoting fibronectin and vimentin synthesis in fibroblasts and keratinocytes (114–116). Additionally, JNK activation of the proliferating cell nuclear antigen (PCNA) and DNA methyltransferase 1 associated protein 1 (DMAP1) domains of DNA methyltransferase 1 (DNMT1) can directly interact with Snail and suppress E-cadherin in colorectal cancer, glioma, and nasopharyngeal carcinoma cell lines (117–119). Furthermore, JNK may be associated with Snail and TWIST1 via c-Jun in multi-drug resistant epidermoid carcinoma and as a downstream effector of Akt in gastric cancer cells (120, 121). Other researches also associated JNK with EMT induction in colorectal cancer (122) and non-small cell lung cancer cells (123). Whereas, for p38 MAPK-mediated EMT, Lin et al. (124) evaluated that p38 MAPK regulated p38 interacting protein (p38IP) and Snail in head and neck squamous cell carcinoma. Another study revealed that p38 MAPK participated in TGF-β induced EMT in glioma cells (125).

To date, there was only one published report on the participation of MAPKs in hypoxia-induced EMT despite MAPKs were found to be important EMT regulators from the view of previous researches. Tam et al. (126) demonstrated that JNK pathway mediates EMT and stemness maintenance of colorectal cancer cells under low oxygen level including hypoxia (1%) and blood oxygen level (10%). This was the first report that showed even the seemingly non-hypoxic 10% oxygen level could affect EMT progression in cancer as that in the traditional hypoxic oxygen level (1–2%). Therefore, MAPK signaling could be an important regulator for hypoxia-induced EMT.

### NF-κB

Nuclear factor κ-light-chain-enhancer of activated B cells (NF-κB) presents in almost all animal cell types and involves in inflammation, immunity, cell proliferation, apoptosis, angiogenesis, tumor metabolism, metastasis, and EMT (127). It can be activated by various stimuli including cytokines, growth factors, radiation, DNA damage and hypoxia (127). NF-κB mediates EMT by cooperating with Ras and TGF-β in breast cancer cells (128). NF-κB was also associated with ezrin and EGF-induced EMT and promoted metastasis in colorectal cancer cells (129). For NF-κB influence on EMT transcription factors, it could directly promote Slug, SIP1, and TWIST1 in breast cancer cells (130). While for the study of hypoxia-induced NF-κB mediated EMT, Cheng et al. (131) concluded that HIF-1α-activated NF-κB could promote EMT in pancreatic cancer cells by inhibiting E-cadherin and promoting N-cadherin. TWIST was promoted by NF-κB but no significant changes of Snail, ZEB1 or ZEB2 were found. Whereas, Kara et al. (132) demonstrated TNF-α-NF-κB axis together with PI3K-Akt axis, contributed to HIF-1α-mediated EMT induction. Therefore, close relationships between NF-κB and HIF-1α may exist in hypoxia-induced EMT.

### Notch

Notch signaling is an evolutionarily conserved pathway which regulates cell differentiation, proliferation and death in all metazoans (133). Notch also involves in tumorigenesis and EMT by the interaction with Delta/Serrate/Lag-2 (DSL) ligands, which subsequently causes a proteolytic cleavage of the Notch receptor protein at the S2 cleavage site with involvement of ADAM10 or ADAM17 (134). Then the second g-secretase-mediated cleavage of the residual part of the Notch protein resulted in the release of the Notch intracellular domain (ICD), which can then directly activate EMT signaling genes (12). For the character of Notch in hypoxia, Notch signaling is important for maintenance of undifferentiated cell state and its intracellular domain cooperates with HIF-1α (135). Notch can directly induce Slug, but not Snail and TWIST1, in breast cancer cell lines (136, 137). However, another study of prostate cancer cells found Notch1 was associated with Snail and ZEB1 (138). Notch can also induce HIF-1α, NF-κB, and miR-200 for EMT induction in various cancer cell lines (139–141). Studies on Notch-mediated hypoxia-induced EMT mainly concentrated on breast cancer cells, which showed Notch worked closely with HIF-1α in hypoxia-induced EMT and the inhibition of Notch could effectively block EMT induction (142, 143). Notch target genes including HES1 and HEY1 were increased by hypoxia and both HIF-1α and HIF-2α synergized with the Notch co-activator MAML1 in promoting Notch activity among breast cancer cells (143). In addition, another study also showed Notch participated in hypoxia-induced EMT in colon cancer, ovarian cancer and glioblastoma with regulation of Snail and HIF-1α (139). Thus, similar to NF-κB, Notch potentiates HIF-1α-induced EMT in hypoxic conditions.

## Future Perspectives

Hypoxia has been conclusively established as a major promoter of EMT. Among various hypoxia-related EMT signaling pathways, HIF-1α is an important mediator of hypoxia-induced EMT in various cancer types. Since HIF-1α is a poor prognosis indicator which also promotes other adverse treatment outcomes, such as chemo and radioresistance, targeting HIF-1α shall increase treatment efficacy and limit metastasis in cancers (144). Inhibiting HIF-1α in different cancer types have found to effectively limit metastasis in both *in vitro* and *in vivo* experiments (145). However, there is a lack of selective HIF-1 inhibitors and clinical trials in this area (145). Thus, the clinical potential of this treatment strategy is yet to be revealed. While for other potential pathways for hypoxia-induced EMT, researches on the characters of these pathways in hypoxic conditions are limited especially for PI3K-Akt-mTOR and MAPKs. Since they have proven roles in mediating EMT induction, exploration of their roles in hypoxia-induced EMT shall provide a better picture for hypoxia-induced EMT. They may be activated in different time period and oxygen tension when compared with HIF-1α. This will be essential for better understanding of metastasis mechanism as metastasis involves complex procedures with tumor cells experiencing different oxygen levels from hypoxia in primary tumor site to blood oxygen level in bloodstream (9). Furthermore, potential therapeutic strategies involving multiple EMT effector inhibitions may effectively inhibit EMT at a wider range of oxygen level, which might reduce tumor metastasis and improve treatment outcome.

## Conclusion

In sum, hypoxia-induced EMT has been established as important route for EMT induction and metastasis in wide range of cancer types *in vitro* and *in vivo*. HIF-1α has proved to be the major mediator of hypoxia-induced EMT. In addition, there are other potential pathways involved in hypoxia-induced EMT which may provide clues for controlling hypoxia-induced EMT by developing new anti-metastatic methods and improving prognosis of cancers.

## Author Contributions

ST and HL contributed in conception and design of the study. ST wrote the original draft. VW and HL supervised the study. HL reviewed and edited the manuscript. All authors read and approved the submitted version.

### Conflict of Interest

The authors declare that the research was conducted in the absence of any commercial or financial relationships that could be construed as a potential conflict of interest.
